# Climate change and suicide epidemiology: a systematic review and meta-analysis of gender variations in global suicide rates

**DOI:** 10.3389/fpubh.2024.1463676

**Published:** 2025-01-08

**Authors:** Dan-Dan Chen, Jin-Heng Tu, Ke-Nan Ling, Xiao-Hong Jin, Hai-Yan Huang

**Affiliations:** ^1^School of Nursing and Rehabilitation, Nantong University, Nantong, China; ^2^Department of Obstetrics and Gynecology, Affiliated Hospital of Nantong University, Nantong, China

**Keywords:** climate change, suicide epidemiology, gender variations, environmental health, suicide risk factors

## Abstract

**Background:**

Climate change is reshaping public health, introducing extreme weather conditions and environmental stressors—such as high temperatures, atmospheric pollution, desertification, and storms (rain, thunder, and hail)—that critically impact mental health. Evidence increasingly links these factors to higher rates of suicide-related outcomes, including suicidal ideation, attempts, and self-harm. Such interactions underscore the importance of understanding how climate-driven mental health risks vary by environmental factor and gender, as gender-specific vulnerabilities shape responses to climate stressors.

**Methods:**

By April 16, 2024, we conducted a comprehensive search of PubMed, Web of Science, Cochrane Library, PsycINFO, Scopus, ProQuest, and Embase. Two researchers independently reviewed studies and collected demographic data, systematically tracking and recording rates of suicidal ideation, suicide attempts, suicide deaths, self-harm, and anxiety. Data were rigorously cross-verified for accuracy and consistency.

**Results:**

The meta-analysis demonstrated significant associations between climate change variables and mental health outcomes. High temperatures and air pollution were linked to increased suicide attempts (OR: 1.40, 95% CI: 1.34–1.45) and suicide deaths (OR: 1.51, 95% CI: 1.44–1.58), particularly among males. Conversely, atmospheric pollution and desertification correlated with a reduced likelihood of suicidal ideation (OR: 0.73, 95% CI: 0.63–0.85). These findings highlight gender-specific mental health impacts, with females exhibiting higher rates of anxiety and self-harm, underscoring the urgent need for targeted interventions addressing climate-induced mental health risks.

**Conclusions:**

This systematic review and meta-analysis reveal significant gender-specific mental health impacts of climate change, with females experiencing higher rates of anxiety, self-harm, and suicidal ideation, while males show greater incidences of suicide attempts and deaths. These findings emphasize the urgent need for targeted interventions and the integration of mental health services into climate policies to address these gender disparities.

**Systematic review registration:**

This study is registered with PROSPERO [PROSPERO (york.ac.uk)] under the identifier [CRD42024534961].

## 1 Introduction

Climate change has emerged as a defining issue of the twenty-first century, with wide-ranging implications for human health and wellbeing. While the physical health impacts of climate change, such as heat-related illnesses and respiratory problems, are well-documented (1, 2), the growing body of evidence highlighting its profound effects on mental health remains underexplored in mainstream discourse (3, 4). Extreme weather events, including heatwaves, floods, and hurricanes, are increasing in both frequency and severity, creating significant psychological stressors that contribute to mental health disorders (5, 6). Among the most concerning mental health outcomes associated with climate change is suicide (7). These outcomes encompass various dimensions, including suicide attempts, suicide deaths, and self-harm, each with distinct implications for public health. Suicide rates are influenced by a complex interplay of factors, including socio-economic status, cultural context, and individual psychological resilience (8, 9). Climate change exacerbates these factors by disrupting social and economic systems, leading to increased stress and mental health challenges (10, 11). For instance, a study by Burke et al. indicated that for every 1°C increase in temperature, the suicide rate can rise by up to 0.7% (12), highlighting the urgent need for understanding the nuanced impacts of climate change on mental health.

Gender differences in suicide rates are well-documented, with men typically having higher rates of suicide completion and women having higher rates of suicide attempts (13, 14). These differences are influenced by a range of biological, psychological, and social factors. Men and women may experience and respond to climate-related stressors differently, necessitating gender-specific approaches to prevention and intervention (15, 16).

Recent research underscores the importance of examining gender variations in mental health outcomes related to climate change. Women, for example, may be more vulnerable to the mental health impacts of climate change due to their often disproportionate exposure to environmental hazards and greater responsibilities for family and community care (17). Additionally, socio-cultural expectations and gender roles can influence how stress and mental health issues are experienced and reported (18, 19). Extreme weather events such as droughts, which affect agricultural productivity, can lead to economic hardship and increased mental health issues, particularly in rural areas. These mental health challenges can manifest in various suicide-related outcomes, including self-harm, suicide attempts, and suicide deaths, which may disproportionately impact women in vulnerable communities. Studies have shown that these impacts are often more severe for women, who may have fewer economic resources and greater caregiving responsibilities (20). In contrast, men may face increased risks of suicide due to societal pressures to be the primary economic providers, which can become overwhelming during times of environmental and economic stress (21, 22).

The intersection of climate change and mental health is a burgeoning field of study, with increasing evidence suggesting that extreme weather events can act as catalysts for mental health crises (23, 24). Heatwaves, in particular, have been closely studied for their impact on mental health. Research has found that high temperatures can lead to increased irritability, aggression, and impulsivity, all of which are risk factors for suicide (25). Furthermore, the chronic stress associated with climate change, such as prolonged droughts and the resultant economic instability, can exacerbate existing mental health conditions and contribute to suicidal behavior (26, 27). Displacement due to natural disasters can also lead to loss of social support networks, increased financial strain, and heightened exposure to traumatic events, all associated with poor mental health outcomes (28, 29). These indirect pathways highlight the need for a holistic approach to understanding and addressing the mental health impacts of climate change.

Gender-specific vulnerabilities to climate change-related mental health issues are shaped by various factors, including differential exposure to environmental hazards, gender roles, and access to resources (30). Women, particularly in low- and middle-income countries, are often disproportionately affected by climate change due to their roles in agriculture, caregiving responsibilities, and other climate-induced livelihood challenges, which can exacerbate mental health vulnerabilities as described by Rosen et al. (31). These roles can increase their exposure to environmental stressors and limit their ability to seek help or relocate in times of crisis. Conversely, men may experience climate change differently, with increased risks of mental health issues stemming from societal expectations of stoicism and self-reliance (32). These expectations can discourage men from seeking mental health support, leading to higher rates of untreated mental health conditions and, consequently, higher suicide rates (33). Understanding these gender-specific responses to climate change is crucial for developing effective public health strategies.

This review aims to provide a comprehensive analysis of the existing literature on the gender-specific impacts of climate change on suicide rates. This study systematically reviews evidence on the relationship between climate change and mental health outcomes, with a specific emphasis on suicide attempts, suicide deaths, and self-harm. By examining these outcomes through the lens of gender differences, we seek to identify patterns that can inform both research and policy. Our goal is to underscore the importance of targeted interventions that address the distinct vulnerabilities of men and women to climate-driven mental health impacts. Overall, this study highlights climate change as a profound and multifaceted challenge to mental health, with notable gender disparities in the manifestation of these effects. Targeted interventions are essential to address the unique vulnerabilities of men and women to the mental health impacts of climate change, contributing to the development of gender-sensitive public health strategies.

## 2 Methods

### 2.1 Search strategy

The systematic review and meta-analysis were conducted in adherence to the Preferred Reporting Items for Systematic Reviews and Meta-Analyses (PRISMA) guidelines (34). Comprehensive literature searches were performed in PubMed, Web of Science, Cochrane Library, PsycINFO, Scopus, ProQuest, and Embase databases, covering publications up to April 16, 2024, without restrictions on publication date. The search strategy utilized both controlled vocabulary and free-text terms related to climate change (e.g., “climate change,” “global warming,” “heat stress”), suicide and self-harm (e.g., “suicide,” “suicidal ideation,” “self-injury”), and gender variations (e.g., “sex characteristics,” “gender differences,” “sexual dimorphism”). Boolean operators were applied to structure the search and improve specificity. Each identified study was screened for relevance, with eligibility independently assessed by two reviewers. An overview of the full search strategy and terms used is provided in Supplementary Table 1.

### 2.2 Eligibility criteria

For inclusion in the systematic review and meta-analysis, studies had to meet the following criteria: (1) The research must have investigated the impact of climate change on suicidal ideation, suicide attempts, suicide deaths, self-harm, and anxiety. Anxiety was included as it has a well-documented link with climate change-related stressors and plays a potential role in exacerbating suicidality (35). Although depression and substance use disorder are also important considerations, anxiety was prioritized in this review due to its frequent occurrence in climate-related mental health studies and its relevance to the gender-specific analysis of mental health vulnerabilities. (2) The studies were required to provide gender-specific data on these outcomes or allow for such data to be derived from secondary sources. (3) Both observational and experimental study designs were considered eligible.

The exclusion criteria were as follows: (1) Studies with incomplete data were excluded. (2) Studies published in languages other than English were not considered. (3) Studies were excluded if the full text was not accessible. (4) Research that combined multiple outcome measures without enabling separate analysis was excluded. (5) In cases of participant overlap across studies, the earliest published study was excluded to prevent data duplication.

### 2.3 Data extraction

To ensure data integrity, two researchers independently extracted the data. Any discrepancies were resolved through consultation with a third reviewer who has expertise in the relevant field. The data extracted from each study included: author name, publication year, study location, recruitment period, and participant characteristics such as age and gender. Outcome measures focused on the prevalence of suicidal ideation, suicide attempts, suicide deaths, self-harm, and anxiety, disaggregated by gender, as well as climate variables such as temperature, atmospheric pollution levels, and desertification indicators, recorded with standardized measurement techniques where applicable. In cases where data were not directly available, we calculated estimates based on secondary sources or inferred values from reported rates to ensure accuracy and consistency. Additionally, we categorized climate variables to facilitate subgroup and sensitivity analyses later in the study. A protocol-guided approach ensured that each stage of data extraction followed a consistent methodology, and our extraction records were carefully documented to enable reproducibility of the findings and enhance transparency in the meta-analytic process.

### 2.4 Statistical analysis

Statistical analyses were performed using Stata (version 11.0 for Windows) to assess the relationship between climate change and mental health outcomes, including suicidal ideation, suicide attempts, suicide deaths, self-harm, and anxiety, by gender. Odds ratios (ORs) with 95% confidence intervals (CIs) were calculated for each outcome. Meta-regression was conducted for suicide deaths and anxiety to examine the influence of key covariates, including weather conditions, study design, gender ratio, country classification, and total sample size. These outcomes were selected for meta-regression based on their homogeneity and the availability of sufficient data. For weather conditions, regions with an average annual temperature above 30°C were categorized as “high temperature” (value = 1), while all other regions were classified as “other” (value = 2). Although specific environmental phenomena such as cold weather, thunderstorms, and rainstorms were evaluated for their impact on mental health outcomes, these were treated separately from the weather category in the meta-regression model.

Due to substantial heterogeneity in measurement methods, sample sizes, and reporting across studies, meta-regression was not performed for other outcomes, such as suicidal ideation, suicide attempts, and self-harm. Subgroup analyses were conducted to explore sources of heterogeneity, categorized by continent, study design, weather exposure type, and gender. Gender-specific subgroup analysis was carried out by grouping studies into male (m1, m2) and female (f1, f2) categories, as shown in Supplementary Figures 1A–5A. Heterogeneity was assessed using the *I*^2^ index and the *Q*-test, while publication bias was examined using funnel plots and Egger's test.

### 2.5 Quality assessment

Each study's quality was evaluated using the Newcastle-Ottawa Scale (36). This scale assesses selection of study groups, comparability between groups, and outcome assessment for cohort studies or exposure for case-control studies. Studies were scored from 0 to 9, with higher scores indicating better quality. Two reviewers independently assessed each study and resolved any differences through discussion. Studies were classified into low, moderate, or high quality based on their scores. Detailed results and classifications are provided in Supplementary Table 2.

### 2.6 Definition of suicide outcomes

Suicide outcomes in this study were classified into four categories: suicidal ideation, suicide attempts, suicide deaths, and self-harm. Suicidal ideation refers to thoughts of self-harm or ending one's life without physical action, representing an early stage of suicide-related behavior and often indicating underlying psychological distress (37, 38). Suicide attempts, in contrast, involve intentional actions aimed at self-harm or death, marking an escalation of suicidal behavior driven by acute emotional pain and external stressors (39–41). Suicide deaths are the final and irreversible outcome of suicide-related behaviors, where the individual deliberately ends their life, typically following prolonged psychological distress or pre-existing mental health conditions (42–44). Self-harm, distinct from the other outcomes, involves deliberate, non-fatal injury to oneself as a maladaptive coping mechanism for emotional turmoil. While self-harm is not driven by a desire to die, it often serves as a temporary release from psychological pain and is frequently seen as a precursor to more severe suicide-related behaviors (45, 46). These definitions provide the basis for the classification and analysis of suicide-related outcomes in this study, ensuring consistency across the results.

## 3 Results

### 3.1 Identification of studies

**Figure 1:**
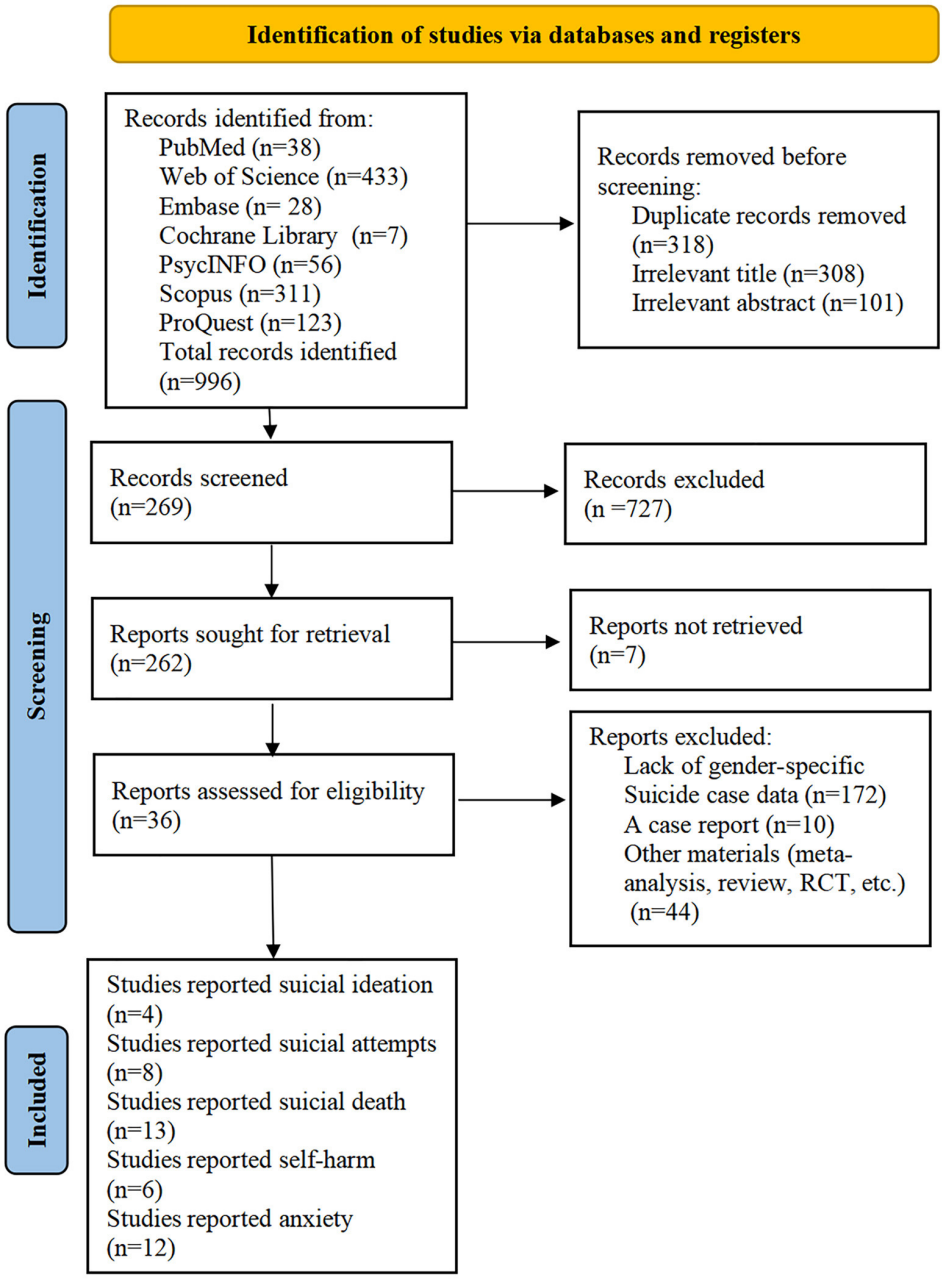
Flowchart depicting the methodology of study selection.

A comprehensive search was conducted across seven databases—PubMed (38), Web of Science (433), Embase (28), Cochrane Library (7), PsycINFO (56), Scopus (311), and ProQuest (123)—yielding a total of 996 studies. After 318 duplicates were removed, 678 unique records remained for screening. Following the title and abstract screening, 409 records were excluded, and 269 articles were selected for full-text review. Further exclusions were made based on factors such as lack of gender-specific suicide data (172), case reports (10), and other irrelevant content, including meta-analyses or reviews (44). In the end, 36 studies met the eligibility criteria and were included in the meta-analysis. The detailed selection process is outlined in Figure 1.

### 3.2 Description of included studies

A total of 36 studies were included in this analysis, conducted across various continents: America, Asia, Europe, Oceania, and Africa. Participants ranged in age, and the studies spanned multiple climate conditions such as high temperatures, atmospheric pollution, and desertification. The studies examined outcomes related to suicidal ideation, suicide attempts, suicide deaths, self-harm, and anxiety. Detailed characteristics of each study, including author, year, study design, demographics, and climate variables, are summarized in Table 1.

**Table 1:** Principal characteristics of the chosen research.

**References**	**Country**	**Period**	**Study design**	**Age**	**Category**	**M(S/N)**	**F(S/N)**	**Environmental phenomenon**	**NOS**
Gunn et al. (78)	Australia	NR	Cross-sectional	49.5 ± 12.3	Anxiety	18/196	19/107	Desertification	5
Nguyen et al. (79)	USA	2005–2013	Retrospective	32.5 ± 19.1	Anxiety	3,639/15,517	4,272/14,909	Atmospheric pollution	8
Nori-Sarma et al. (80)	USA	2010–2019	Cross–sectional	51.0 ± 18.8	Anxiety	256/968	518/1,511	High temperature	5
Tawatsupa et al. (81)	Thailand	2005	Cross-sectional	51.0 ± 36.0	Anxiety	1,341/18,148	2,051/22,765	High temperature	6
Thomas et al. (82)	India	2018–2019	Cross-sectional	41.5 ± 23.5	Anxiety	19/34	38/80	Rainstorm	6
Ndetei et al. (83)	Kenya	2022	Cross-sectional	18.0 ± 5.0	Anxiety	526/1,728	286/862	Atmospheric pollution	7
Hanigan et al. (84)	Australia	2015	Cross-sectional	47.0 ± 29.0	Anxiety	678/2,205	1,135/3,592	Desertification	7
Basu et al. (85)	USA	2005–2013	Retrospective	43.0 ± 18.4	Anxiety	14,158/30,778	17,298/31,578	Atmospheric pollution	7
Di Nicola et al. (86)	Italy	2016–2019	Cross-sectional	44.0 ± 26.0	Anxiety	37/130	51/142	Cold	7
Gunn et al. (87)	Australia	2008	Cross-sectional	54.5 ± 30.5	Anxiety	7/28	18/72	Desertification	6
Sewell et al. (88)	USA	2016–2019	Cross-sectional	14.2 ± 4.1	Anxiety	434/1,122	688/1,515	Desertification	7
Mellado et al. (89)	Chile	2017	Cross-sectional	14.5 ± 2.1	Anxiety	16/124	21/137	Thunderstorms	7
Salib et al. (90)	UK	1989–1993	Retrospective	46.0 ± 18.0	Self–harm	29/152	9/42	Thunderstorms	7
Kubo et al. (91)	Japan	2012–2015	Retrospective	37.5 ± 13.8	Self-harm	925/5,505	1,811/9,099	High temperature	7
Bjorksten et al. (92)	Denmark	1968–1995	Retrospective	47.5 ± 21.4	Self-harm	112/684	30/149	High temperature	8
Nguyen et al. (79)	USA	2005–2013	Retrospective	32.5 ± 19.1	Self-harm	1,279/7,935	2,284/11,418	Atmospheric pollution	8
Nori-Sarma et al. (80)	USA	2010–2019	Cross-sectional	51.0 ± 18.8	Self-harm	174/968	277/1,318	High temperature	5
Basu et al. (85)	USA	2005–2013	Retrospective	43.0 ± 18.4	Self-harm	2,116/13,222	3,996/19,026	Atmospheric pollution	7
Viswanathan et al. (93)	India	2017	Cross-sectional	45.0 ± 25.0	Suicidal ideation	38/124	39/70	Desertification	5
Ndetei et al. (83)	Kenya	2022	Cross-sectional	18.0 ± 5.0	Suicidal ideation	235/1,728	156/862	Atmospheric pollution	7
Brokamp et al. (94)	USA	2011–2015	Cross-sectional	15.0 ± 12.8	Suicidal ideation	48/112	66/163	Atmospheric pollution	8
Sewell et al. (88)	USA	2016-2019	Cross-sectional	14.2 ± 4.1	Suicidal ideation	103/803	233/1,233	Desertification	7
Ambar et al. (95)	France	2009–2018	Retrospective	NR	Suicide attempts	3,672/27,483	2,409/25,063	Cold	8
Dumencic et al. (96)	Croatia	2000–2011	Retrospective	NR	Suicide attempts	213/329	356/718	Cold	8
Hiltunen et al. (97)	Finland	1989–1990	Cross-sectional	NR	Suicide attempts	202/1,198	104/1,111	Atmospheric pollution	5
Ndetei et al. (83)	Kenya	2022	Cross-sectional	18.0 ± 5.0	Suicide attempts	74/525	67/602	Atmospheric pollution	7
Szyszkowicz et al. (98)	USA	1999–2003	Cross-sectional	35.0 ± 5.0	Suicide attempts	390/1,042	145/563	Atmospheric pollution	7
Lee et al. (99)	Korea	2005–2022	Cross-sectional	49.4 ± 15.6	Suicide attempts	3,219/20,323	1,132/10,381	Atmospheric pollution	6
Yarza et al. (100)	Palestine	2002–2017	Cross-sectional	41.6 ± 20.7	Suicide attempts	164/1,008	201/1,330	High temperature	6
Miyazaki et al. (101)	Japan	2005–2022	Cross-sectional	41.1 ± 17.4	Suicide attempts	281/773	297/964	Atmospheric pollution	7
Lee et al. (102)	Korea	2002–2013	Retrospective	NR	Suicide deaths	72/494	26/247	Atmospheric pollution	6
Kim et al. (103)	Korea	2001–2005	Retrospective	NR	Suicide deaths	843/5,777	271/3,113	High temperature	8
Dumencic et al. (96)	Croatia	2000–2011	Retrospective	NR	Suicide deaths	82/569	43/478	Cold	8
Kok et al. (104)	China	1980–1989	Retrospective	30.0 ± 17.6	Suicide deaths	224/1,690	112/1,199	High temperature	8
Hiltunen et al. (105)	Finland	1969–2003	Retrospective	NR	Suicide deaths	858/6,597	621/6,889	High temperature	7
Hanigan et al. (106)	Australia	1971–1999	Prospective	30.0 ± 20.0	Suicide deaths	1,053/7,256	681/7,235	Desertification	6
Law et al. (107)	Australia	1996–2007	Cross-sectional	NR	Suicide deaths	450/3,023	180/2,165	Cold	6
Lehmann et al. (108)	France	1968–2016	Retrospective	NR	Suicide deaths	263/1,836	188/1,901	High temperature	5
Beautrais et al. (109)	New Zeeland	2007–2015	Retrospective	NR	Suicide deaths	54/169	2/16	Desertification	6
Partonen et al. (110)	Finland	1987–1988	Cross-sectional	76.5 ± 12.5	Suicide deaths	98/793	21/248	Cold	6
Hiltunen et al. (111)	Finland	1979–2010	Retrospective	NR	Suicide deaths	771/5,756	244/2,517	High temperature	6
Qin et al. (112)	Denmark	1982–2013	Retrospective	60.5 ± 19.5	Suicide deaths	1,237/8,753	475/4,947	Atmospheric pollution	6
Luo et al. (113)	China	2013–2018	Retrospective	NR	Suicide deaths	1,256/8,547	404/4,075	Cold	8

NR stands for “not applicable”; NOS refers to the “Newcastle-Ottawa Scale,” used for evaluating the quality of non-randomized studies included in meta-analyses; S/N represents the ratio of males or females exhibiting suicidal ideation, suicide attempts, suicide deaths, or self-harm, or experiencing anxiety, to the number of men or women exposed to extreme weather conditions.

### 3.3 Weather variables classification

This section critically examines the associations between climate variables—high temperature, atmospheric pollution, desertification, and other extreme weather phenomena—and mental health outcomes, including suicidal ideation, suicide attempts, suicide deaths, self-harm, and anxiety. By disaggregating results by climate variable and considering geographic differences, this analysis highlights the distinct impacts of these environmental factors on suicide-related outcomes and anxiety. Supplementary Figures support the presentation of pooled estimates and subgroup analyses.

#### 3.3.1 High temperature

High temperature emerged as a significant climate variable, influencing mental health outcomes in complex ways. Meta-analysis results (Supplementary Figure 1A) revealed a significant inverse association between high temperature and suicidal ideation (OR: 0.73, 95% CI: 0.63–0.85, *I*^2^ = 28.1%). This trend was particularly observed in regions such as Kenya and India, where prolonged exposure to high temperatures may have led to physiological acclimatization and psychological adaptation, potentially reducing the prevalence of suicidal ideation. Conversely, high temperature was strongly associated with an increased risk of suicide attempts (OR: 1.08, 95% CI: 0.86–1.34, *I*^2^ = 0.0%, Supplementary Figure 2D) and suicide deaths (OR: 1.48, 95% CI: 1.38–1.58, *I*^2^ = 1.3%, Supplementary Figure 3D). These associations were most pronounced in Oceania (Supplementary Figure 3B), where recurrent heatwaves, inadequate infrastructure, and limited adaptive capacity likely amplified vulnerabilities, particularly in populations with pre-existing mental health conditions or limited access to healthcare. Anxiety demonstrated an unexpected protective relationship with high temperatures (OR: 0.81, 95% CI: 0.76–0.87, *I*^2^ = 0.0%, Supplementary Figure 5D). The available data on self-harm related to high temperatures were limited, with most studies showing no significant effect.

#### 3.3.2 Atmospheric pollution

Atmospheric pollution emerged as a significant environmental stressor, strongly associated with adverse mental health outcomes, especially in urban and industrialized regions. The meta-analysis (Supplementary Figure 2D) indicated a positive association between atmospheric pollution and suicide attempts (OR: 1.44, 95% CI: 1.35–1.53, *I*^2^ = 49.5%), with the highest risks observed in densely populated areas of Asia and North America, where prolonged exposure to fine particulate matter (PM2.5) is common. A similar association was identified for suicide deaths (OR: 1.47, 95% CI: 1.32–1.64, *I*^2^ = 0.0%, Supplementary Figure 3D), likely mediated by neuroinflammatory and oxidative stress mechanisms. Anxiety showed an unexpected inverse relationship with atmospheric pollution (OR: 0.84, 95% CI: 0.82–0.86, *I*^2^ = 0.8%, Supplementary Figure 5D). This inverse trend may reflect contextual factors such as healthcare access, cultural differences in symptom recognition, or regional reporting practices.

#### 3.3.3 Desertification

Desertification was uniquely associated with mental health outcomes, often showing protective trends. The meta-analysis (Supplementary Figure 1A) revealed a significant inverse association between desertification and suicidal ideation (OR: 0.73, 95% CI: 0.63–0.85, *I*^2^ = 28.1%). This protective effect was most pronounced in desertification-prone regions such as Kenya and India, where long-term exposure to arid conditions likely fostered community-based coping mechanisms and enhanced social support networks. Desertification also exhibited a protective trend for anxiety (OR: 0.92, 95% CI: 0.84–1.00, Supplementary Figure 5D), though this finding should be interpreted with caution due to study design variability and potential reporting biases.

#### 3.3.4 Other weather phenomena

Other weather phenomena, including cold weather, thunderstorms, and rainstorms, were evaluated for their impact on mental health outcomes. The meta-analysis results from Supplementary Figures 2D, 3D, 5D revealed significant associations between these weather events and mental health conditions. Cold weather was positively associated with suicide attempts (OR: 1.38, 95% CI: 1.31–1.46, Supplementary Figure 2D) and suicide deaths (OR: 1.57, 95% CI: 1.43–1.72, Supplementary Figure 3D), indicating an increased risk of these severe outcomes during colder conditions. This may reflect exacerbated mental health conditions or region-specific factors, such as limited social support and healthcare infrastructure during cold spells. Additionally, cold weather was inversely associated with anxiety (OR: 0.79, 95% CI: 0.49–1.29, Supplementary Figure 5D), suggesting that lower temperatures might be associated with reduced anxiety, potentially due to decreased external stressors or coping mechanisms that arise in colder environments.

Thunderstorms demonstrated a protective effect on anxiety (OR: 0.84, 95% CI: 0.42–1.69, Supplementary Figure 5D), with anxiety levels potentially reduced during thunderstorms. This effect may be due to the predictability of these weather events, allowing individuals to mentally prepare and reducing the uncertainty and anxiety often associated with unexpected environmental changes. Rainstorms, on the other hand, showed an association with anxiety (OR: 1.18, 95% CI: 0.60–2.33, Supplementary Figure 5D), which was somewhat less clear, indicating that the impact of rainstorms on mental health may be more context-dependent, possibly influenced by social support systems and local coping mechanisms in affected areas.

### 3.4 Suicide outcomes classification

This section delves into the associations between distinct suicide-related outcomes—suicidal ideation, suicide attempts, suicide deaths, self-harm, and anxiety—and climate change variables. By systematically analyzing results for each outcome and incorporating findings from sensitivity tests, subgroup analyses, and meta-regression, it seeks to unravel the unique pathways through which environmental factors shape these mental health outcomes. Supplementary Figures are provided to substantiate the robustness and reliability of the observed associations.

#### 3.4.1 Suicidal ideation

The pooled analysis demonstrated a significant inverse association between suicidal ideation and specific climate-related factors, with an overall OR of 0.73 (95% CI: 0.63–0.85) and moderate heterogeneity (*I*^2^ = 28.1%). These findings suggest that suicidal ideation may be influenced by environmental conditions such as desertification and atmospheric pollution, which appear to foster protective trends in certain contexts. These effects could be linked to long-term adaptation mechanisms, including strengthened community networks and psychological resilience, that help mitigate the psychological burden associated with chronic exposure to environmental stressors.

The robustness of these results was confirmed through sensitivity analysis (Supplementary Figure 1B), which showed no significant changes in the pooled OR when individual studies were excluded. Additionally, the symmetrical funnel plot (Supplementary Figure 1C) and Egger's test (*p* = 0.244) indicated no evidence of publication bias, reinforcing confidence in the observed associations.

#### 3.4.2 Suicide attempts

Our meta-analysis revealed a significant association between climate change variables and suicide attempts, with a pooled OR of 1.40 (95% CI: 1.34–1.45) and moderate heterogeneity (*I*^2^ = 51.1%, *p* = 0.046; Supplementary Figure 2A). Subgroup analyses indicated geographic variability: studies conducted in Asia reported a pooled OR of 1.39 (95% CI: 1.30–1.48) with high heterogeneity (*I*^2^ = 78.8%, *p* = 0.009), while European studies showed a similar OR of 1.40 (95% CI: 1.33–1.47) but with moderate heterogeneity (*I*^2^ = 54.2%, *p* = 0.112; Supplementary Figure 2B).

Retrospective studies demonstrated a pooled OR of 1.38 (95% CI: 1.31–1.46) with minimal heterogeneity (*I*^2^ = 0.0%, *p* = 0.579), while cross-sectional studies had a slightly higher OR of 1.41 (95% CI: 1.33–1.49) with increased heterogeneity (*I*^2^ = 64.0%, *p* = 0.016; Supplementary Figure 2C). Sensitivity analysis (Supplementary Figure 2E) confirmed the stability of these results, with consistent OR values across individual study exclusions. The funnel plot (Supplementary Figure 2F) displayed symmetry, and Egger's test (*p* = 0.246) indicated no significant publication bias, further reinforcing the reliability of the findings.

#### 3.4.3 Suicide deaths

Our meta-analysis revealed a significant association between climate change variables and suicide deaths, with a pooled OR of 1.51 (95% CI: 1.44–1.58) and no observed heterogeneity (*I*^2^ = 0.0%, *p* = 0.704; Supplementary Figure 3A). Subgroup analyses revealed geographic variations: studies conducted in Asia reported an OR of 1.54 (95% CI: 1.41–1.67), while in Europe, the OR was slightly lower at 1.45 (95% CI: 1.36–1.54). Oceania exhibited the highest OR of 1.60 (95% CI: 1.47–1.75; Supplementary Figure 3B). Analysis by study design showed that retrospective studies demonstrated a pooled OR of 1.48 (95% CI: 1.41–1.56), prospective studies yielded a similar OR of 1.54 (95% CI: 1.39–1.71), while cross-sectional studies showed a significantly higher OR of 1.75 (95% CI: 1.47–2.07; Supplementary Figure 3C).

Sensitivity analysis (Supplementary Figure 3E) confirmed the robustness of these results, with no significant changes in the pooled OR after excluding individual studies. The symmetrical funnel plot (Supplementary Figure 3F) and Egger's test (*p* = 0.704) showed no evidence of publication bias, supporting the reliability of the findings.

Meta-regression analysis results (Table 2) provided further insight into the factors influencing the association between climate variables and suicide deaths. Gender ratio emerged as the only significant predictor of suicide deaths (B = 0.013, *p* = 0.046), suggesting that a higher proportion of males in the population is associated with an increased risk of suicide mortality in the presence of climate stressors. This finding indicates that gender-specific vulnerabilities may play a critical role in moderating the effects of environmental stressors on suicide mortality. Other factors, such as study year (B = 0.002, *p* = 0.487), sample size (B = −0.00001, *p* = 0.753), and country development status (B = −0.003, *p* = 0.672), were not significantly associated with the outcome, indicating that these factors may not substantially influence the observed associations between climate variables and suicide deaths.

**Table 2:** Meta-regression analysis table for suicide deaths.

**Variables**	**B**	**SE**	**95% Cl**	***t***	***p***
Year	0.002	0.002	[−0.002, 0.006]	1.000	0.320
Gender ratio	0.013	0.005	[−0.001, 0.027]	2.600	0.046
Study period midpoint	0.003	0.002	[−0.001, 0.007]	1.500	0.154
Country (developed)	0.016	0.025	[−0.033, 0.065]	0.640	0.530
Country (developing)	0.011	0.021	[−0.030, 0.052]	0.520	0.610
Weather category (high temperature)	−0.021	0.036	[−0.093, 0.051]	−0.583	0.569
Weather category (other)	0.006	0.031	[−0.055, 0.067]	0.194	0.847
Study design (cross–sectional)	−0.009	0.041	[−0.089, 0.071]	−0.220	0.828
Study design (retrospective)	0.011	0.051	[−0.089, 0.111]	0.220	0.827
Total sample size	−0.002	0.002	[−0.006, 0.002]	−1.000	0.330

#### 3.4.4 Self-harm

Our meta-analysis revealed a significant inverse association between climate change variables and self-harm, with a pooled OR of 0.79 (95% CI: 0.76–0.83) and no observed heterogeneity (*I*^2^ = 0.0%, *p* = 0.456; Supplementary Figure 4A). This consistent finding suggests that exposure to certain climate-related factors, such as high temperatures or atmospheric pollution, may be associated with a reduced risk of self-harm.

The stability and robustness of these results were confirmed through sensitivity analysis (Supplementary Figure 4B), where no significant changes in the pooled OR were observed upon exclusion of individual studies. Furthermore, the symmetrical funnel plot (Supplementary Figure 4C) and Egger's test (*p* = 0.437) provided no evidence of publication bias, reinforcing the reliability of the results. Given the absence of heterogeneity and the stability of the findings, meta-regression analysis was not conducted, as it would likely provide limited additional insights.

#### 3.4.5 Anxiety

The meta-analysis revealed a significant inverse association between climate change variables and anxiety, with a pooled odds ratio (OR) of 0.84 (95% CI: 0.82–0.86) and low heterogeneity (*I*^2^ = 19.1%, *p* = 0.256; Supplementary Figure 5A). This suggests that exposure to certain climate factors, such as elevated temperatures and atmospheric pollution, is associated with reduced anxiety levels in certain contexts.

Subgroup analyses showed geographic variations. Studies conducted in Oceania reported an OR of 0.96 (95% CI: 0.86–1.07), with moderate heterogeneity (*I*^2^ = 37.2%, *p* = 0.203). In contrast, research from America showed a more consistent association, with an OR of 0.83 (95% CI: 0.81–0.85) and no heterogeneity (*I*^2^ = 0.0%, *p* = 0.793). Studies from Asia, Africa, and Europe yielded ORs ranging from 0.79 to 0.92, with minimal heterogeneity, indicating that the relationship between climate factors and anxiety is relatively stable across these regions (Supplementary Figure 5B). Regarding study design, retrospective studies yielded a pooled OR of 0.83 (95% CI: 0.82–0.85) with no heterogeneity (*I*^2^ = 0.0%, *p* = 0.373). Cross-sectional studies showed a slightly higher OR of 0.86 (95% CI: 0.82–0.90), with moderate heterogeneity (*I*^2^ = 24.5%, *p* = 0.218; Supplementary Figure 5C). This suggests that retrospective studies may reflect more consistent long-term trends, whereas cross-sectional studies might capture the immediate, context-specific impacts of climate factors on anxiety.

Sensitivity analysis confirmed the robustness of these findings (Supplementary Figure 5E), with stable ORs observed even after excluding individual studies. The symmetrical funnel plot (Supplementary Figure 5F) and Egger's test (*p* = 0.356) indicated no evidence of publication bias, further supporting the reliability of these results.

Meta-regression analysis (Table 3) was conducted to explore potential predictors of the association between climate factors and anxiety outcomes. The analysis revealed that the gender ratio was the only variable significantly associated with anxiety outcomes (B = 0.014, *p* = 0.039). Specifically, an increased male-to-female ratio within the study populations was linked to a more pronounced inverse relationship between climate stressors and anxiety. This suggests that gender-specific factors, such as coping strategies and vulnerability to climate-related stressors, may significantly influence anxiety outcomes. In contrast, other variables, including study year (B = 0.002, *p* = 0.487), sample size (B = −0.00001, *p* = 0.753), and country development status (B = −0.003, *p* = 0.672), were not found to significantly impact the observed relationship between climate change variables and anxiety. These findings underscore the complexity of the interaction between climate factors and anxiety, highlighting the potential role of gender in modulating these associations, while other demographic and methodological variables appear to have minimal influence.

**Table 3:** Meta-regression analysis table for anxiety.

**Variables**	**B**	**SE**	**95% Cl**	***t***	***p***
Year	0.001	0.002	[−0.003, 0.005]	0.500	0.625
Gender ratio	0.014	0.006	[−0.002, 0.030]	2.333	0.039
Study period midpoint	0.004	0.003	[−0.002, 0.010]	1.333	0.203
Country (developed)	0.017	0.026	[−0.034, 0.068]	0.654	0.525
Country (developing)	0.012	0.022	[−0.031, 0.055]	0.545	0.590
Weather category (high temperature)	−0.022	0.037	[−0.096, 0.052]	−0.595	0.559
Weather category (other)	0.007	0.032	[−0.057, 0.071]	0.219	0.828
Study design (cross-sectional)	−0.010	0.042	[−0.094, 0.074]	−0.238	0.823
Study design (retrospective)	0.012	0.052	[−0.092, 0.116]	0.231	0.823
Total sample size	−0.003	0.003	[−0.007, 0.001]	−1.000	0.330

## 4 Discussion

The discussion highlights critical insights into how climate change impacts mental health differently across genders, with specific focus on suicidal ideation, suicide attempts, suicide deaths, self-harm, and anxiety. The findings suggest that climate change intensifies mental health disparities, with women experiencing heightened risks of anxiety, self-harm, and suicidal ideation, likely due to unique social and caregiving pressures during climate-induced crises. In contrast, men are more prone to suicide attempts and deaths, potentially driven by societal expectations of stoicism and the economic stress associated with their roles as primary providers. These gender-specific responses underscore the importance of understanding how climate-related stressors interact with socio-cultural factors to influence mental health outcomes.

### 4.1 Climate variables

The impact of high temperature on mental health is dual-faceted, with both protective and harmful effects observed across different regions. In areas such as Kenya and India, where populations have adapted to prolonged heat exposure, high temperatures were inversely associated with suicidal ideation, likely due to enhanced social resilience and coping mechanisms. However, in regions like Oceania, recurrent heatwaves, combined with poor infrastructure and limited healthcare access, exacerbated mental health vulnerabilities, leading to an increased risk of suicide attempts and deaths. These findings emphasize the importance of context in shaping the relationship between temperature and mental health outcomes, suggesting that adaptive strategies can mitigate risks in certain regions, while other areas require targeted public health interventions to enhance resilience.

Atmospheric pollution, particularly in urban and industrialized regions, emerged as a significant environmental stressor with detrimental effects on mental health. Prolonged exposure to pollutants, such as PM2.5, was associated with an increased risk of suicide attempts and deaths, possibly through neuroinflammatory and oxidative stress mechanisms that exacerbate psychological vulnerability. While anxiety showed an inverse relationship with atmospheric pollution, this unexpected finding may reflect regional differences in healthcare access, reporting biases, or cultural norms influencing symptom recognition. These results underline the need for policies aimed at reducing air pollution and improving urban living conditions to mitigate the mental health impacts of poor air quality.

Desertification, although less impactful than other climate variables, demonstrated protective effects in regions prone to arid conditions, such as Kenya and India. In these areas, community-based coping mechanisms and enhanced social support networks appear to buffer against psychological distress, leading to a significant inverse association with suicidal ideation and anxiety. However, the mental health impact of desertification on more severe outcomes like suicide attempts and deaths remains poorly understood, with limited data available for robust analysis. Further research is needed to explore the long-term psychological consequences of desertification, particularly in underrepresented regions, to guide interventions that strengthen community resilience and mental health support.

Extreme weather events, including thunderstorms, hailstorms, and rainstorms, had more context-dependent impacts on mental health outcomes. While thunderstorms and hailstorms were linked to increased impulsivity and suicide attempts, the effects of rainstorms were more variable, likely due to regional differences in flood severity and disaster preparedness. Extreme weather events tend to disrupt both environmental and social systems, leading to acute psychological distress. These findings highlight the need for localized interventions to strengthen disaster response systems and mental health support in regions vulnerable to such events. Particularly in areas with inadequate disaster management or low socioeconomic resilience, extreme weather can exacerbate existing mental health conditions, underscoring the importance of context in determining the mental health impact of climate phenomena.

### 4.2 Suicidal ideation

Our meta-analysis incorporated data from four studies, encompassing 2,767 male and 2,328 female participants, to assess the prevalence of suicidal ideation in the context of climate change. The results revealed a higher prevalence of suicidal ideation among females, 21.2% (95% CI: 19.59–22.90), compared to males, 15.3% (95% CI: 13.99–16.66). This suggests that females may be more susceptible to the psychological stressors associated with climate change. This gender disparity aligns with existing literature, which often attributes higher rates of mental health issues in females to factors such as increased caregiving responsibilities and greater economic vulnerabilities during extreme weather events (47). Moreover, the pooled odds ratio for suicidal ideation related to climate change was 0.73 (95% CI: 0.63–0.85), underscoring a significant inverse association with specific environmental factors such as desertification and atmospheric pollution. These findings suggest that long-term exposure to these stressors may foster certain protective mechanisms, such as enhanced psychological resilience or community-level adaptations, particularly in regions like India or urban centers in the USA and Kenya (48, 49).

The mechanisms underlying these associations remain complex and multifactorial. Gender-specific differences, including biological susceptibility to stress, sociocultural roles, and access to coping resources, may contribute to these disparities. Furthermore, unlike suicide attempts or deaths, suicidal ideation is less influenced by acute external stressors and may instead reflect cumulative psychological burdens exacerbated by prolonged exposure to climate stressors. These distinctions highlight the necessity of tailoring preventive strategies to address the unique pathways through which climate factors influence suicidal ideation, ensuring interventions are sensitive to both gender and regional vulnerabilities.

### 4.3 Suicide attempts

Our analysis, encompassing data from eight studies with 52,681 male and 40,732 female participants, revealed that suicide attempts were more prevalent among males, at 15.6% (95% CI: 15.28–15.91), compared to females, at 11.6% (95% CI: 11.26–11.88). This gender disparity highlights the distinct ways in which men and women respond to climate-related stress. Men, often subjected to societal pressures and cultural norms emphasizing stoicism and economic responsibility, may be less likely to express emotions or seek mental health support. Consequently, they are more prone to extreme responses, such as suicide attempts, under severe stress (50). This aligns with broader mental health research, which suggests that men's reluctance to seek help or share emotional burdens can lead to the accumulation of unaddressed psychological distress, culminating in drastic actions. Conversely, women are more likely to report mental health issues, such as anxiety and depression, but these may not translate into suicide attempts or deaths at the same rate. Women's coping mechanisms, which often involve seeking social support and engaging in emotional expression, may serve as protective factors against transitioning from ideation to attempts (51). This distinction underscores the need to address gender-specific pathways in understanding suicide attempts. Climate-induced stressors, such as economic instability, loss of livelihood, and displacement, may disproportionately impact men. The traditional expectation for men to be primary economic providers intensifies the psychological burden of these challenges, potentially exacerbating their vulnerability to suicide attempts. Meanwhile, women's higher rates of suicidal ideation may reflect chronic psychological distress, but their coping strategies and access to stronger social support networks may act as a buffer, preventing the progression to attempts (52, 53).

### 4.4 Suicide deaths

Data from 13 studies involving 51,260 male and 35,029 female participants revealed a higher prevalence of suicide deaths among males, at 14.2% (95% CI: 13.86–14.67), compared to females, at 9.3% (95% CI: 9.02–9.63). This gender disparity aligns with well-documented epidemiological trends, wherein men are more likely to complete suicide than women.

The heightened risk for males may be attributed to societal norms that emphasize economic provision and stoicism. During climate-induced crises, such as economic instability or resource scarcity, these expectations can intensify psychological stress, potentially leading to an increased likelihood of suicide deaths (54). Men may also experience barriers to seeking emotional or psychological support, contributing to the accumulation of unaddressed distress and a higher risk of fatal outcomes. Additionally, the physiological effects of extreme climate events, such as heatwaves, which exacerbate mental health conditions, may further amplify risks among vulnerable male populations.

In contrast, women, while facing significant psychological burdens due to climate change, often exhibit lower suicide death rates. This may be partly explained by their higher likelihood of seeking social and psychological support, as well as their reliance on established coping mechanisms during crises (55). Women's caregiving roles, however, introduce unique stressors, as they are frequently tasked with ensuring family safety and wellbeing during extreme weather events or periods of environmental degradation. These responsibilities can lead to heightened anxiety, emotional distress, and burnout (56). Furthermore, women frequently serve as central figures in disaster response and recovery efforts, both formally and informally, exposing them to trauma and the emotional suffering of others. While this exposure increases their risk of anxiety and depression, it may also enable them to provide emotional support to others, creating a dual role that acts as both a stressor and a protective factor (57).

These findings underscore the complex interplay of gender, societal expectations, and climate-induced stressors in shaping suicide death outcomes. For men, fostering a culture that encourages emotional expression and provides accessible mental health resources may help reduce the risk of suicide deaths. For women, strengthening support networks and addressing the added caregiving and emotional burdens imposed by climate crises are essential for mitigating mental health challenges.

### 4.5 Self-harm

Our analysis of six studies involving 28,466 male and 41,052 female participants revealed a higher prevalence of self-harm among females, 20.5% (95% CI: 20.09–20.87), compared to males, 16.3% (95% CI: 15.85–16.71). This gender difference likely reflects variations in emotional regulation and social expectations. Women, often carrying the dual burdens of caregiving and societal pressures, may internalize climate-induced stressors differently from men, leading to heightened rates of self-harm. In contrast, males, who are more prone to externalizing behaviors under stress, exhibit higher rates of suicide attempts and deaths, indicating a divergence in behavioral responses to similar environmental triggers.

Climate-induced factors, such as extreme heat, play a prominent role in self-harm. Unlike suicidal behaviors, which may be driven by cumulative despair, self-harm is often linked to acute triggers, such as physiological stress and heightened impulsivity during heatwaves (58). Prolonged exposure to high temperatures exacerbates emotional dysregulation and diminishes coping reserves, increasing vulnerability to self-harming acts. These patterns differ from the broader existential despair observed in suicide deaths, where chronic environmental degradation and socioeconomic instability are often primary drivers (59). Rapid weather changes, including abrupt temperature shifts or prolonged rainfall, disrupt daily routines and intensify feelings of instability. For individuals with pre-existing mental health conditions, this instability amplifies psychological distress, triggering self-harm behaviors. Unlike suicide attempts, which often reflect a direct response to acute crises, self-harm can arise from persistent emotional dysregulation fostered by unpredictable climate patterns (60). Seasonal patterns further illuminate the distinct relationship between self-harm and climate change. Seasonal affective disorder (SAD), linked to reduced sunlight in winter, is associated with depressive episodes that elevate self-harm risk (61). As climate change alters seasonal cycles, the severity and timing of SAD-related self-harm may intensify, marking a divergence from other suicide-related outcomes that are less influenced by seasonal variability (62). This highlights the need for context-specific mental health interventions that address seasonal impacts.

In summary, self-harm represents a unique behavioral response to climate stressors, distinct from suicidal ideation, attempts, and deaths in its etiology and manifestation. While self-harm often serves as a maladaptive coping strategy for managing emotional distress, its prevalence and triggers differ significantly across genders and environmental contexts.

### 4.6 Anxiety

The inclusion of anxiety in this analysis reflects its critical role in understanding the full spectrum of climate change's impact on mental health. Unlike suicide-related outcomes, which are often the culmination of extreme psychological distress, anxiety represents an early and pervasive response to environmental stressors. By addressing anxiety as a distinct yet interconnected mental health outcome, this study highlights its significance as both a standalone condition and a precursor to more severe outcomes, such as suicidal ideation and attempts. This approach ensures a comprehensive examination of climate change's multifaceted effects on mental wellbeing.

The analysis, incorporating data from 12 studies with 70,978 male and 77,270 female participants, reveals a higher prevalence of anxiety among females (34.2%; 95% CI: 33.83–34.49) compared to males (29.8%; 95% CI: 29.43–30.11). This gender disparity aligns with existing literature suggesting that women, who often bear greater caregiving responsibilities and face heightened socio-economic vulnerabilities, are disproportionately affected by climate-related stressors (63). These findings underscore the importance of addressing gender-specific dynamics in mental health responses to climate change.

Anxiety's relationship with climate change is mediated by various pathways. Extreme weather events, such as hurricanes, floods, and wildfires, trigger immediate psychological responses, including fear and heightened alertness, which can persist as chronic anxiety about future occurrences. This sustained hypervigilance, often referred to as “disaster anxiety,” can escalate into generalized anxiety disorder if unaddressed (64). Additionally, temperature extremes exacerbate anxiety symptoms, with high temperatures contributing to physical discomfort and health issues like dehydration, while low temperatures increase isolation and reduce social interaction, both of which amplify psychological distress (65). “Eco-anxiety,” a term describing chronic anxiety about environmental degradation, further exemplifies the profound impact of climate change on mental health (66). This condition manifests as persistent worry about the planet's future, driven by witnessing environmental destruction and the perceived inadequacy of responses to climate challenges (67). For individuals with strong connections to nature, the degradation of natural environments represents a profound emotional loss, intensifying feelings of helplessness and despair. This unique form of anxiety, while distinct, often intersects with broader mental health outcomes by eroding resilience and increasing vulnerability to severe psychological conditions.

Social and economic disruptions caused by climate change, such as rising sea levels, agricultural failures, and competition for resources, further exacerbate anxiety levels. Vulnerable populations, particularly those in poverty or with limited access to resources, are disproportionately affected, experiencing heightened uncertainty and chronic stress (68). These socio-economic stressors not only amplify anxiety but also create conditions that may lead to more severe outcomes, such as suicidal ideation or attempts (69).

By contextualizing anxiety within the broader framework of climate change-induced mental health outcomes, this analysis emphasizes its interconnectedness with suicide-related behaviors. Anxiety often serves as a gateway condition, with chronic, unmanaged distress potentially escalating to self-harm or suicide in vulnerable individuals. Addressing anxiety at this early stage offers an opportunity to intervene and mitigate progression to more severe psychological outcomes, reinforcing the importance of including anxiety as a critical focus in climate-related mental health research and interventions.

### 4.7 Mitigating climate-induced mental health crises

Addressing the mental health impacts of climate change requires a comprehensive approach, integrating prevention and intervention strategies to mitigate the associated risks of suicidal ideation, suicide attempts, suicide deaths, self-harm, and anxiety. Early identification and regular screening of at-risk populations are essential. Healthcare providers should employ standardized screening tools during routine medical visits and community health programs to detect early signs of mental health issues (70). Ensuring accessible mental health services, especially in regions most affected by climate change, is critical. Telemedicine can extend the reach of counseling and psychiatric care to remote or underserved areas, offering timely support (71). Strengthening community resilience through social cohesion programs and mutual support initiatives, such as peer support groups and mental health workshops, can significantly aid individuals in coping with climate-induced psychological stressors (72). Public education campaigns are necessary to raise awareness about the mental health impacts of climate change and the importance of seeking help. These campaigns should aim to destigmatize mental health issues and encourage proactive management. Integrating mental health considerations into climate policies and disaster response plans ensures that mental health services are included in emergency relief and long-term recovery efforts (73). Healthcare providers require specialized training to address the mental health impacts of climate change effectively. This training should cover trauma-informed care and the specific needs of climate-affected populations. Gender-specific interventions are also crucial, as women and men experience different mental health impacts from climate change. Support programs tailored to these needs can significantly reduce the rates of anxiety, self-harm, and suicidal behavior. Providing economic support and reducing social vulnerabilities can alleviate the stress exacerbated by climate change, thus mitigating mental health crises. Policies that offer financial assistance, job security, and housing stability are essential (17, 74, 75). Engaging communities in environmental stewardship activities can enhance mental wellbeing by fostering a sense of purpose and connection (76). Lastly, developing robust crisis intervention strategies, including hotlines, mobile crisis units, and rapid response teams, ensures immediate support during climate-related emergencies (77).

## 5 Limitations

This study presents a systematic review of the global literature on the mental health impacts of climate change, highlighting significant gender-specific differences. The methodology is rigorous, covering a wide range of databases and strictly adhering to PRISMA guidelines to ensure the inclusion of high-quality studies. Nevertheless, several limitations should be acknowledged. First, reliance on self-reported data may introduce biases, as individuals could underreport or over report mental health symptoms. Additionally, the variability in climate conditions and socio-economic contexts across different regions may affect the generalizability of the findings. A significant limitation of this study is the exclusion of non-English publications, potentially overlooking relevant research from certain regions where climate change impacts and mental health responses may differ. Furthermore, there is considerable heterogeneity in outcomes related to suicidal ideation, suicide attempts, and self-harm, largely due to variations in definitions and measurement methods across studies. This heterogeneity could lead to inconsistencies in the observed mental health impacts of climate change. Lastly, while the gender-specific analysis provides important insights, this review does not fully explore other demographic factors, such as age, socio-economic status, and cultural background, which also shape mental health responses to climate change.

## 6 Conclusions

This systematic review and meta-analysis highlight the significant gender-specific mental health impacts of climate change. Our findings indicate that females are more likely to experience anxiety, self-harm, and suicidal ideation, while males show higher rates of suicide attempts and deaths. These gender disparities reflect distinct psychological responses to climate stressors. Females, who often bear additional caregiving responsibilities and are more vulnerable economically during extreme weather events, appear to be more susceptible to chronic psychological distress. In contrast, males, influenced by societal expectations to be stoic and financially responsible, exhibit higher rates of extreme behaviors, such as suicide attempts and deaths, under acute stress.

The results underscore the need for targeted mental health interventions that consider these gender-specific vulnerabilities. While both genders are deeply affected by climate change, their coping mechanisms and the risks they face differ considerably. Future research should continue to focus on the mechanisms behind these gender differences, particularly the role of societal norms, caregiving burdens, and access to mental health resources in shaping responses to climate-induced stress. Addressing these differences will be essential in crafting effective mental health strategies in the face of ongoing climate change.

## Data Availability

The original contributions presented in the study are included in the article/Supplementary material, further inquiries can be directed to the corresponding author.

## Supplementary material

Supplementary Figure 1 Meta-analysis of suicidal ideation: **(A)** Forest diagram illustrating the aggregated odds ratios; **(B)** Sensitivity analysis chart for the included research; **(C)** Funnel diagram to assess potential publication biases.

Supplementary Figure 2 Meta-analysis of Suicide attempts: **(A)** Forest diagram illustrating the aggregated odds ratios; **(B)** Forest plot of combined odds ratios (analyzed by continent); **(C)** Forest plot of combined odds ratios (analyzed by study design); **(D)** Forest plot of combined odds ratios (analysis by type of weather); **(E)** Sensitivity analysis chart for the included research; **(F)** Funnel diagram to assess potential publication biases.

Supplementary Figure 3 Meta-analysis of suicide deaths: **(A)** Forest diagram illustrating the aggregated odds ratios; **(B)** Forest plot of combined odds ratios (analyzed by continent); **(C)** Forest plot of combined odds ratios (analyzed by study design); **(D)** Forest plot of combined odds ratios (analysis by type of weather); **(E)** Sensitivity analysis chart for the included research; **(F)** Funnel diagram to assess potential publication biases.

Supplementary Figure 4 Meta-analysis of self-harm: **(A)** Forest diagram illustrating the aggregated odds ratios; **(B)** Sensitivity analysis chart for the included research; **(C)** Funnel diagram to assess potential publication biases.

Supplementary Figure 5 Meta-analysis of anxiety: **(A)** Forest diagram illustrating the aggregated odds ratios; **(B)** Forest plot of combined odds ratios (analyzed by continent); **(C)** Forest plot of combined odds ratios (analyzed by study design); **(D)** Forest plot of combined odds ratios (analysis by type of weather); **(E)** Sensitivity analysis chart for the included research; **(F)** Funnel diagram to assess potential publication biases.

Supplementary Table 1 Search strategy.

Supplementary Table 2 Newcastle-Ottawa scale.
