# Supplementary material for: Climate change and suicide epidemiology: a systematic review and meta-analysis of gender variations in global suicide rates
Source: Front Public Health. 2025 Jan 8;12:1463676. doi: 10.3389/fpubh.2024.1463676 (PMC11750776; doi:10.3389/fpubh.2024.1463676)
Supplement: Supplementary Figure 1 — Meta-analysis of suicidal ideation: (A) Forest diagram illustrating the aggregated odds ratios; (B) Sensitivity analysis chart for the included research; (C) Funnel diagram to assess potential publication biases. [file Table_3.doc]

Supplementary Figure 1 Meta-analysis of suicidal ideation: A. Forest diagram illustrating the aggregated odds ratios; B. Sensitivity analysis chart for the included research; C. Funnel diagram to assess potential publication biases.


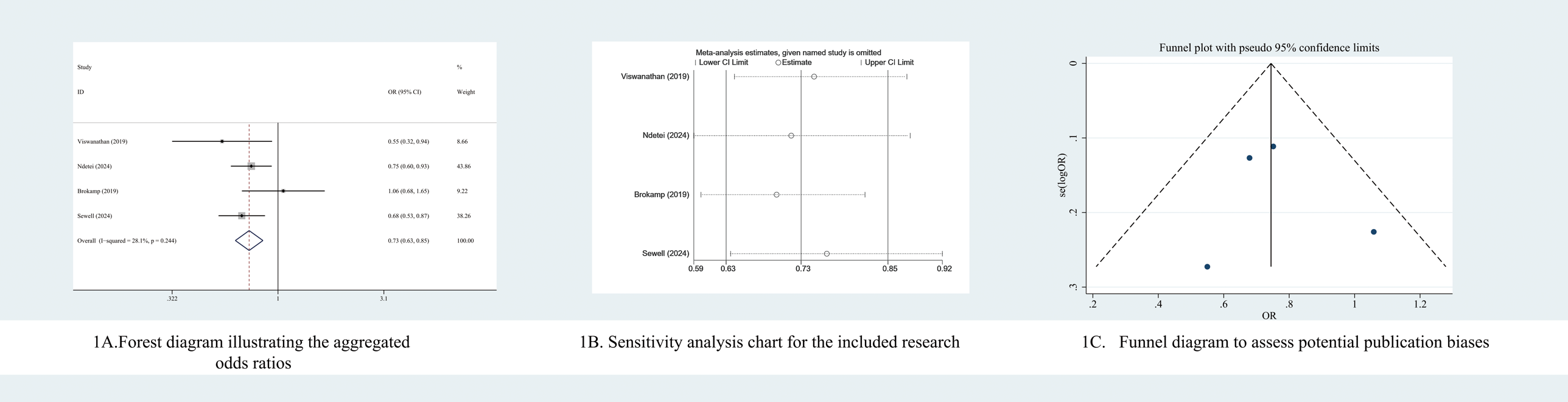


Note: To view the full-sized chart, please right-click on the image and select the "Preview" option.

Supplementary Figure 2 Meta-analysis of Suicide attempts: A. Forest diagram illustrating the aggregated odds ratios; B. Forest plot of combined odds ratios (analyzed by continent); C. Forest plot of combined odds ratios (analyzed by study design); D. Forest plot of combined odds ratios (analysis by type of weather); E. Sensitivity analysis chart for the included research; F. Funnel diagram to assess potential publication biases.





Note: To view the full-sized chart, please right-click on the image and select the "Preview" option.

Supplementary Figure 3 Meta-analysis of suicide deaths:A. Forest diagram illustrating the aggregated odds ratios; B. Forest plot of combined odds ratios (analyzed by continent); C. Forest plot of combined odds ratios (analyzed by study design); D. Forest plot of combined odds ratios (analysis by type of weather); E. Sensitivity analysis chart for the included research; F. Funnel diagram to assess potential publication biases.





Note: To view the full-sized chart, please right-click on the image and select the "Preview" option.

Supplementary Figure 4 Meta-analysis of self-harm:A. Forest diagram illustrating the aggregated odds ratios; B. Sensitivity analysis chart for the included research; C. Funnel diagram to assess potential publication biases.


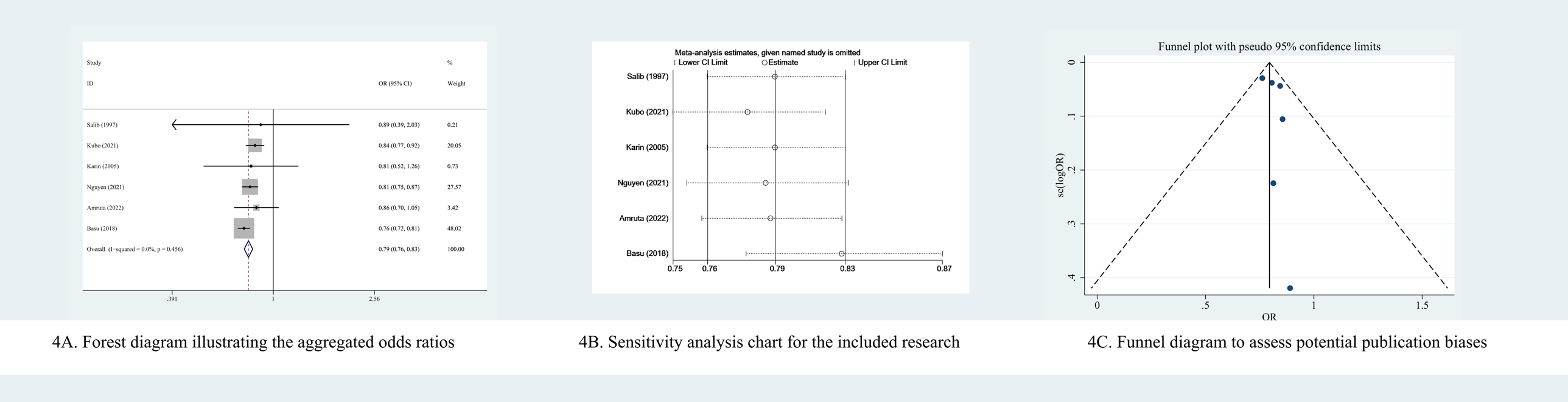


Note: To view the full-sized chart, please right-click on the image and select the "Preview" option.

Supplementary Figure 5 Meta-analysis of anxiety:A. Forest diagram illustrating the aggregated odds ratios; B. Forest plot of combined odds ratios (analyzed by continent); C. Forest plot of combined odds ratios (analyzed by study design); D. Forest plot of combined odds ratios (analysis by type of weather); E. Sensitivity analysis chart for the included research; F. Funnel diagram to assess potential publication biases.





Note: To view the full-sized chart, please right-click on the image and select the "Preview" option.
