# Supplementary material for: Climate change and suicide epidemiology: a systematic review and meta-analysis of gender variations in global suicide rates
Source: Front Public Health. 2025 Jan 8;12:1463676. doi: 10.3389/fpubh.2024.1463676 (PMC11750776; doi:10.3389/fpubh.2024.1463676)
Supplement: Supplementary Table 1 — Search strategy. [file Table_1.docx]

Table S2.Datebase search strategy.

1. Pubmed Datebase search strategy

| Search number | Query | Results |
| --- | --- | --- |
| 10 | #3 and #6 and #9 | 38 |
| 9 | #7 or #8 | 2,473,314 |
| 8 | ((((((((((((((((((((((((((((((((gender difference) OR (Sex Characteristics)) OR (Genders)) OR (Male)) OR (Female)) OR (Man)) OR (Woman)) OR (Boy)) OR (girl)) OR (Characteristic, Sex)) OR (Sex Characteristic)) OR (Sexual Dimorphism)) OR (Dimorphism, Sexual)) OR (Sexual Dimorphisms)) OR (Gender Differences)) OR (Gender Difference)) OR (Sex Dimorphism)) OR (Dimorphism, Sex)) OR (Sex Dimorphisms)) OR (Gender Characteristics)) OR (Characteristic, Gender)) | 2,451,608 |
| 7 | "Sex Characteristics"[Mesh] | 62,306 |
| 6 | #4 or #5 | 89,541 |
| 5 | (((((((((((((Suicide) OR (Suicidal Ideation)) OR (Suicide Prevention)) OR (Suicide, Assisted)) OR (Suicide, Attempted)) OR (Suicide, Completed)) OR (suicide attempt)) OR (Suicide mortality rate)) OR (self-injury)) OR (suicide program)) OR (Non-suicidal self-injury)) OR (suicidal behavior)) OR (self-injury behavior)))) | 36,328 |
| 4 | "Suicide"[Mesh] | 76,895 |
| 3 | #1 or #2 | 158,813 |
| 2 | (((((((((((((((((climate change) OR (Change, Climate)) OR (Changes, Climate)) OR (Climate Changes)) OR (Global Warming)) OR (Climat)) OR (heat stress)) OR (ambient temperature)) OR (Humidity)) OR (Rainfall)) OR (extreme weather)) OR (Extreme cold)) OR (greenhouse gases)) OR (Greenhouse Gas)) OR (Gas, Greenhouse)) OR (Droughts)) OR (Desert Climate))) | 135,262 |
| 1 | "Climate Change"[Mesh] | 32,358 |

Date Run:2024/4/16

1. web of science Database search strategy

| #1 | climate change (Topic) or Change, Climate (Topic) or Changes, Climate (Topic) or Climate Changes (Topic) or Global Warming (Topic) or Climat (Topic) or heat stress (Topic) or ambient temperature (Topic) or Humidity (Topic) or Rainfall (Topic) or extreme weather (Topic) or Extreme cold (Topic) or greenhouse gases (Topic) or Greenhouse Gas (Topic) or Gas, Greenhouse (Topic) or Droughts (Topic) or Desert Climate (Topic) and Preprint Citation Index (Exclude – Database) | 34,867 |
| --- | --- | --- |
| #2 | Suicide (Topic) or Suicidal Ideation (Topic) or Suicide Prevention (Topic) or Suicide, Assisted (Topic) or Suicide, Attempted (Topic) or Suicide, Completed (Topic) or suicide attempt (Topic) or Suicide mortality rate (Topic) or self-injury (Topic) or suicide program (Topic) or Non-suicidal self-injury (Topic) or self-injury behavior (Topic) and Preprint Citation Index (Exclude – Database) | 36,689 |
| #3 | Sex Characteristics (Topic) or gender difference (Topic) or Genders (Topic) or Male (Topic) or Female (Topic) or Man (Topic) or Woman (Topic) or Boy (Topic) or girl (Topic) or Characteristic, Sex (Topic) or Sex Characteristic (Topic) or Sexual Dimorphism (Topic) or Dimorphism, Sexual (Topic) or Sexual Dimorphisms (Topic) or Gender Differences (Topic) or Gender Difference (Topic) or Sex Dimorphism (Topic) or Dimorphism, Sex (Topic) or Sex Dimorphisms (Topic) or Gender Characteristics (Topic) or Characteristic, Gender (Topic) or Gender Characteristic (Topic) or Gender Dimorphism (Topic) or Dimorphism, Gender (Topic) or Gender Dimorphisms (Topic) or Sex Differences (Topic) or Difference, Sex (Topic) or Sex Difference (Topic) or Sexual Dichromatism (Topic) or Dichromatism, Sexual (Topic) or Dichromatisms, Sexual (Topic) or Sexual Dichromatisms (Topic) and Preprint Citation Index (Exclude – Database) | 233,140 |
| #4 | #1 AND #2 AND #3 and Preprint Citation Index (Exclude – Database) | 433 |

Date Run:2024/4/16

1. PsycINFO Database search strategy

| No. | Query | Results |
| --- | --- | --- |
| #4 | #1 AND #2 AND #3 | 56 |
| #3 | TX Sex Characteristics OR TX gender difference OR TX Genders OR TX Male OR TX Female OR TX Man OR TX Woman OR TX Boy OR TX girl OR TX Characteristic, Sex OR TX Sex Characteristic OR TX Sexual Dimorphism OR TX Dimorphism, Sexual OR TX Sexual Dimorphisms OR TX Gender Differences OR TX Gender Difference OR TX Sex Dimorphism OR TX Dimorphism, Sex OR TX Sex Dimorphisms OR TX Gender Characteristics OR TX Characteristic, Gender OR TX Gender Characteristic OR TX Gender DimorphismOR TX Dimorphism, Gender OR TX Gender Dimorphisms OR TX Sex Differences OR TX Difference, Sex OR TX Sex Difference OR TX Sexual Dichromatism OR TX Dichromatism, Sexual OR TX Dichromatisms, Sexual OR TX Sexual Dichromatisms | 2,128,343 |
| #2 | TX Suicidal Ideation OR TX Suicide Prevention OR TX Suicide, Assisted OR TX Suicide, Attempted OR TX Suicide, Completed OR TX Suicide OR TX suicide attempt OR TX Suicide mortality rate OR TX self-injury OR TX suicide program OR TX Non-suicidal OR TX suicidal behavior OR TX self-injury behavior | 65,564 |
| #1 | TX climate change OR TX Change, Climate OR TX Changes, Climate OR TX Climate Changes OR TX Climat OR TX Global Warming OR TX heat stress OR TX ambient temperature OR TX Humidity OR TX Rainfall OR TX extreme weather OR TX Extreme cold OR TX greenhouse gases OR TX Greenhouse Gas OR TX Gas, Greenhouse OR TX Droughts OR TX Desert Climate | 9,365 |

Date Run:2024/4/16

1. Scopus Database search strategy

| #1 | ( TITLE-ABS-KEY-AUTH ( climate AND change ) OR TITLE-ABS-KEY-AUTH ( change, AND climate ) OR TITLE-ABS-KEY-AUTH ( changes, AND climate ) OR TITLE-ABS-KEY-AUTH ( climate AND changes ) OR TITLE-ABS-KEY-AUTH ( global AND warming ) OR TITLE-ABS-KEY-AUTH ( climat ) OR TITLE-ABS-KEY-AUTH ( heat AND stress ) OR TITLE-ABS-KEY-AUTH ( ambient AND temperature ) OR TITLE-ABS-KEY-AUTH ( rainfall ) OR TITLE-ABS-KEY-AUTH ( extreme AND weather ) OR TITLE-ABS-KEY-AUTH ( humidity ) OR TITLE-ABS-KEY-AUTH ( extreme AND cold ) OR TITLE-ABS-KEY-AUTH ( greenhouse AND gases ) OR TITLE-ABS-KEY-AUTH ( greenhouse AND gas ) OR TITLE-ABS-KEY-AUTH ( gas, AND greenhouse ) OR TITLE-ABS-KEY-AUTH ( droughts ) OR TITLE-ABS-KEY-AUTH ( desert AND climate ) ) | 1,681,944 |
| --- | --- | --- |
| #2 | ( TITLE-ABS-KEY-AUTH ( suicide ) OR TITLE-ABS-KEY-AUTH ( suicidal AND ideation ) OR TITLE-ABS-KEY-AUTH ( suicide AND prevention ) OR TITLE-ABS-KEY-AUTH ( suicide, AND assisted ) OR TITLE-ABS-KEY-AUTH ( suicide, AND attempted ) OR TITLE-ABS-KEY-AUTH ( suicide, AND completed ) OR TITLE-ABS-KEY-AUTH ( suicide AND attempt ) OR TITLE-ABS-KEY-AUTH ( suicide AND mortality AND rate ) OR TITLE-ABS-KEY-AUTH ( self-injury ) OR TITLE-ABS-KEY-AUTH ( suicide AND program ) OR TITLE-ABS-KEY-AUTH ( non-suicidal AND self-injury ) OR TITLE-ABS-KEY-AUTH ( suicidal AND behavior ) OR TITLE-ABS-KEY-AUTH ( self-injury AND behavior ) OR TITLE-ABS-KEY-AUTH ( self-injury ) ) | 184,225 |
| #3 | ( TITLE-ABS-KEY-AUTH ( sex AND characteristics ) OR TITLE-ABS-KEY-AUTH ( gender AND difference ) OR TITLE-ABS-KEY-AUTH ( genders ) OR TITLE-ABS-KEY-AUTH ( male ) OR TITLE-ABS-KEY-AUTH ( female ) OR TITLE-ABS-KEY-AUTH ( man ) OR TITLE-ABS-KEY-AUTH ( woman ) OR TITLE-ABS-KEY-AUTH ( boy ) OR TITLE-ABS-KEY-AUTH ( girl ) OR TITLE-ABS-KEY-AUTH ( characteristic, AND sex ) OR TITLE-ABS-KEY-AUTH ( sex AND characteristic ) OR TITLE-ABS-KEY-AUTH ( sexual AND dimorphism ) OR TITLE-ABS-KEY-AUTH ( dimorphism, AND sexual ) OR TITLE-ABS-KEY-AUTH ( sexual AND dimorphisms ) OR TITLE-ABS-KEY-AUTH ( gender AND differences ) OR TITLE-ABS-KEY-AUTH ( gender AND difference ) OR TITLE-ABS-KEY-AUTH ( sex AND dimorphism ) OR TITLE-ABS-KEY-AUTH ( dimorphism, AND sex ) OR TITLE-ABS-KEY-AUTH ( sex AND dimorphisms ) OR TITLE-ABS-KEY-AUTH ( gender AND characteristics ) OR TITLE-ABS-KEY-AUTH ( characteristic, AND gender ) OR TITLE-ABS-KEY-AUTH ( gender AND characteristic ) OR TITLE-ABS-KEY-AUTH ( gender AND dimorphism ) OR TITLE-ABS-KEY-AUTH ( dimorphism, AND gender ) OR TITLE-ABS-KEY-AUTH ( gender AND dimorphisms ) OR TITLE-ABS-KEY-AUTH ( sex AND differences ) OR TITLE-ABS-KEY-AUTH ( difference, AND sex ) OR TITLE-ABS-KEY-AUTH ( sex AND difference ) OR TITLE-ABS-KEY-AUTH ( sexual AND dichromatism ) OR TITLE-ABS-KEY-AUTH ( dichromatism, AND sexual ) OR TITLE-ABS-KEY-AUTH ( dichromatisms, AND sexual ) OR TITLE-ABS-KEY-AUTH ( sexual AND dichromatisms ) ) | 17,294,136 |
| #4 | #1 AND #2 AND #3 | 311 |

Date Run:2024/4/16

1. ProQuest Database search strategy

| #1 | summary(climate change) OR summary(Change, Climate) OR summary(Changes, Climate) OR summary(Climate Changes) OR summary(Global Warming) OR summary(Climat) OR summary(heat stress) OR summary(ambient temperature) OR summary(Humidity) OR summary(Rainfall) OR summary(extreme weather) OR summary(Extreme cold) OR summary(greenhouse gases) OR summary(Greenhouse Gas) OR summary(Gas, Greenhouse) OR summary(Droughts) OR summary(Desert Climate) | 784,017 |
| --- | --- | --- |
| #2 | summary(Suicide) OR summary(Suicidal Ideation) OR summary(Suicide Prevention) OR summary(Suicide, Assisted) OR summary(Suicide, Attempted) OR summary(Suicide, Completed) OR summary(suicide attempt) OR summary(Suicide mortality rate) OR summary(self-injury) OR summary(suicide program) OR summary(Non-suicidal self-injury) OR summary(suicidal behavior) OR summary(self-injury behavior) | 142,402 |
| #3 | summary(Sex Characteristics) OR summary(gender difference) OR summary(Genders) OR summary(Male) OR summary(Female) OR summary(Man) OR summary(Woman) OR summary(Boy) OR summary(girl) OR summary(Characteristic, Sex) OR summary(Sex Characteristic) OR summary(Sexual Dimorphism) OR summary(Dimorphism, Sexual) OR summary(Sexual Dimorphisms) OR summary(Gender Differences) OR summary(Gender Difference) OR summary(Sex Dimorphism) OR summary(Dimorphism, Sex) OR summary(Sex Dimorphisms) OR summary(Gender Characteristics) OR summary(Characteristic, Gender) OR summary(Gender Characteristic) OR summary(Gender Dimorphism) OR summary(Dimorphism, Gender) OR summary(Gender Dimorphisms) OR summary(Sex Differences) OR summary(Difference, Sex) OR summary(Sexual Dichromatism) OR summary(Dichromatism, Sexual) OR summary(Dichromatisms, Sexual) OR summary(Sexual Dichromatisms) | 5,477,627 |
| #4 | #1 AND #2 AND #3 | 123 |

Date Run:2024/4/16

1. Cochrane Database search strategy

| No. | Query | Results |
| --- | --- | --- |
| #10 | #3 AND #6 AND #9 | 7 |
| #9 | #7 OR #8 |  |
| #8 | (Suicidal Ideation) OR (Suicide Prevention) OR (Suicide, Assisted) OR (Suicide, Attempted) OR (Suicide, Completed) OR (Suicide) OR (suicide attempt) OR (Suicide mortality rate) OR (self-injury) OR (suicide program) OR (Non-suicidal self-injury) OR (suicidal behavior) OR (self-injury behavior) | 7,786 |
| #7 | MeSH descriptor: [Suicide] explode all trees | 2,129 |
| #6 | #4 OR #5 |  |
| #5 | Sex Characteristics OR gender difference OR Genders OR Male OR Female OR Man OR Woman OR Boy OR girl OR Characteristic, Sex OR Sex Characteristic OR Sexual Dimorphism OR Dimorphism, Sexual OR Sexual Dimorphisms OR Gender Differences OR Gender Difference OR Sex Dimorphism OR Dimorphism, Sex OR Sex Dimorphisms OR Gender Characteristics OR Characteristic, Gender OR Gender Characteristic OR Gender DimorphismOR Dimorphism, Gender OR Gender Dimorphisms OR Sex Differences OR Difference, Sex OR Sex Difference OR Sexual Dichromatism OR Dichromatism, Sexual OR Dichromatisms, Sexual OR Sexual Dichromatisms | 1,156,771 |
| #4 | MeSH descriptor: [Sex Characteristics] explode all trees | 1,652 |
| #3 | #1 OR #2 |  |
| #2 | climate change OR Change, Climate OR Changes, Climate OR Climate Changes OR Climat OR Global Warming OR heat stress OR ambient temperature OR Humidity OR Rainfall OR extreme weather OR Extreme cold OR greenhouse gases OR Greenhouse Gas OR Gas, Greenhouse OR Droughts OR Desert Climate | 5,517 |
| #1 | MeSH descriptor: [Climate Change] explode all trees | 43 |

Date Run:2024/4/16

1. Embase Datebase search strategy

| No. | Query | Results |
| --- | --- | --- |
| #4 | #1 AND #2 AND #3 | 28 |
| #3 | (gender difference) OR (Sex Characteristics) OR (Genders) OR (Male) OR (Female)OR (Man) OR (Woman) OR (Boy)OR (girl) OR (Characteristic, Sex)OR (Sex Characteristic) OR (Sexual Dimorphism)OR (Dimorphism, Sexual) OR (Sexual Dimorphisms) OR (Gender Differences) OR (Gender Difference)OR (Sex Dimorphism) OR (Dimorphism, Sex) OR (Sex Dimorphisms) OR (Gender Characteristics) OR (Characteristic, Gender) | 125,351 |
| #2 | (Suicide) OR (Suicidal Ideation) OR (Suicide Prevention) OR (Suicide, Assisted)OR (Suicide, Attempted) OR (Suicide, Completed)OR (suicide attempt) OR (Suicide mortality rate) OR (self-injury) OR (suicide program)OR (Non-suicidal self-injury)OR (suicidal behavior) OR (self-injury behavior) | 7,534 |
| #1 | (climate change) OR (Change, Climate) OR (Changes, Climate)OR (Climate Changes)OR (Global Warming)OR (Climat) OR (heat stress) OR (ambient temperature) OR (Humidity) OR (Rainfall) OR (extreme weather)OR (Extreme cold) OR (greenhouse gases) OR (Greenhouse Gas) OR (Gas, Greenhouse) OR (Droughts) OR (Desert Climate) | 5,140 |

Date Run:2024/4/16
