# Supplementary material for: Climate change and suicide epidemiology: a systematic review and meta-analysis of gender variations in global suicide rates
Source: Front Public Health. 2025 Jan 8;12:1463676. doi: 10.3389/fpubh.2024.1463676 (PMC11750776; doi:10.3389/fpubh.2024.1463676)
Supplement: Supplementary Table 2 — Newcastle-Ottawa scale. [file Table_2.docx]

| Study | Selection |  |  |  | Comparability | Exposure |  |  | Scores |
| --- | --- | --- | --- | --- | --- | --- | --- | --- | --- |
|  | Adequate definition of cases | Representat iveness of the cases | Selection of con trols | Definition of con trols | Control for important factor | Ascertain-ment of exposure | Same method of ascertain-ment for cases and contrels | Non-response rate |  |
| Salib et al.,1997 | ☆ | ☆ |  | ☆ | ☆ | ☆ | ☆ | ☆ | 7 |
| Ambar et al.,2022 | ☆ | ☆ | ☆ |  | ☆☆ | ☆ | ☆ | ☆ | 8 |
| Kubo et al.,2021 | ☆ | ☆ | ☆ |  | ☆☆ | ☆ | ☆ |  | 7 |
| Lee et al.,2018 | ☆ |  | ☆ |  | ☆☆ | ☆ |  | ☆ | 6 |
| Gunn et al.,2012 | ☆ |  | ☆ |  | ☆☆ | ☆ |  |  | 5 |
| Yoonhee et al.,2011 | ☆ | ☆ | ☆ | ☆ | ☆ | ☆ | ☆ | ☆ | 8 |
| Karin et al.,2005 | ☆ | ☆ | ☆ | ☆ | ☆☆ | ☆ | ☆ |  | 8 |
| Dumencic et al.,2019 | ☆ | ☆ | ☆ | ☆ | ☆ | ☆ | ☆ | ☆ | 8 |
| Nguyen et al.,2021 | ☆ | ☆ | ☆ | ☆ | ☆ | ☆ | ☆ | ☆ | 8 |
| Kok et al.,1993 | ☆ | ☆ | ☆ | ☆ | ☆ | ☆ | ☆ | ☆ | 8 |
| Viswanathan et al.,2019 | ☆ |  | ☆ |  | ☆☆ | ☆ |  |  | 5 |
| Hiltunen et al.,2012 | ☆ |  | ☆ |  | ☆☆ | ☆ | ☆ | ☆ | 7 |
| Amruta et al.,2022 | ☆ |  | ☆ |  | ☆☆ | ☆ |  |  | 5 |
| Hanigan et al.,2022 | ☆ | ☆ | ☆ |  | ☆☆ | ☆ |  |  | 6 |
| Chi-kin et al.,2013 | ☆ |  | ☆ | ☆ | ☆☆ | ☆ |  |  | 6 |
| Hiltunen et al.,2011 | ☆ |  | ☆ |  | ☆☆ | ☆ |  |  | 5 |
| Tawatsupa et al.,2010 | ☆ |  | ☆ |  | ☆☆ | ☆ | ☆ |  | 6 |
| Thomas et al.,2021 | ☆ |  | ☆ |  | ☆☆ | ☆ |  | ☆ | 6 |
| Ndetei et al.,2024 | ☆ | ☆ |  | ☆ | ☆☆ | ☆ | ☆ |  | 7 |
| Lehmann et al.,2022 | ☆ |  | ☆ |  | ☆ | ☆ | ☆ |  | 5 |
| Hanigan et al.,2018 | ☆ |  | ☆ | ☆ | ☆☆ | ☆ | ☆ |  | 7 |
| Basu et al.,2018 | ☆ | ☆ |  |  | ☆☆ | ☆ | ☆ | ☆ | 7 |
| Beautrais et al.,2018 | ☆ | ☆ |  | ☆ | ☆ | ☆ | ☆ |  | 6 |
| Partonen et al.,2004 | ☆ | ☆ |  |  | ☆☆ | ☆ |  | ☆ | 6 |
| Hiltunen et al.,2014 | ☆ | ☆ | ☆ |  | ☆☆ | ☆ |  |  | 6 |
| Qin et al.,2013 | ☆ | ☆ |  |  | ☆☆ | ☆ |  | ☆ | 6 |
| Brokamp et al.,2019 | ☆ | ☆ | ☆ |  | ☆☆ | ☆ | ☆ | ☆ | 8 |
| Di Nicola et al.,2020 | ☆ | ☆ | ☆ |  | ☆☆ | ☆ | ☆ |  | 7 |
| Kate et al.,2021 | ☆ | ☆ |  |  | ☆☆ | ☆ |  |  | 6 |
| Sewell et al.,2024 | ☆ | ☆ | ☆ |  | ☆☆ | ☆ |  | ☆ | 7 |
| Szyszkowicz et al.,2010 | ☆ | ☆ |  |  | ☆☆ | ☆ | ☆ | ☆ | 7 |
| Hyewon et al.,2019 | ☆ | ☆ |  | ☆ | ☆ | ☆ |  | ☆ | 6 |
| Miyazaki et al.,2023 | ☆ | ☆ | ☆ | ☆ | ☆ | ☆ |  | ☆ | 7 |
| Liying et al.,2019 | ☆ | ☆ | ☆ | ☆ | ☆ | ☆ | ☆ | ☆ | 8 |
| Yarza et al.,2020 | ☆ | ☆ | ☆ |  | ☆ | ☆ |  | ☆ | 6 |
| Mellado et al.,2022 | ☆ | ☆ | ☆ |  | ☆☆ | ☆ | ☆ |  | 7 |
